# Analytical Determination of Serotonin Exocytosis in Human Platelets with BDD-on-Quartz MEA Devices

**DOI:** 10.3390/bios14020075

**Published:** 2024-01-31

**Authors:** Rosalía González Brito, Pablo Montenegro, Alicia Méndez, Ramtin E. Shabgahi, Alberto Pasquarelli, Ricardo Borges

**Affiliations:** 1 Pharmacology Unit, Medical School, Universidad de La Laguna, 38200 La Laguna, Spain; rgonzalb@ull.edu.es (R.G.B.); pmontene@ull.edu.es (P.M.); alu0101025445@ull.edu.es (A.M.); 2 Institute of Electron Devices and Circuits, Ulm University, 89069 Ulm, Germany; ramtin.eghbal-shabgahi@uni-ulm.de (R.E.S.); alberto.pasquarelli@uni-ulm.de (A.P.)

**Keywords:** amperometry, electrochemistry, exocytosis, serotonin, human platelets

## Abstract

Amperometry is arguably the most widely used technique for studying the exocytosis of biological amines. However, the scarcity of human tissues, particularly in the context of neurological diseases, poses a challenge for exocytosis research. Human platelets, which accumulate 90% of blood serotonin, release it through exocytosis. Nevertheless, single-cell amperometry with encapsulated carbon fibers is impractical due to the small size of platelets and the limited number of secretory granules on each platelet. The recent technological improvements in amperometric multi-electrode array (MEA) devices allow simultaneous recordings from several high-performance electrodes. In this paper, we present a comparison of three MEA boron-doped diamond (BDD) devices for studying serotonin exocytosis in human platelets: (i) the BDD-on-glass MEA, (ii) the BDD-on-silicon MEA, and (iii) the BDD on amorphous quartz MEA (BDD-on-quartz MEA). Transparent electrodes offer several advantages for observing living cells, and in the case of platelets, they control activation/aggregation. BDD-on-quartz offers the advantage over previous materials of combining excellent electrochemical properties with transparency for microscopic observation. These devices are opening exciting perspectives for clinical applications.

## 1. Introduction

Exocytosis is the cellular mechanism that through the fusion of intracellular organelles with the plasma membrane, allows the release of internal content to the outside. It is a discrete or quantum process, where the molecules stored inside vesicles, granules, lysosomes, or other cellular structures are released as chemical packages [1,2,3,4]. Exocytosis is the primary mechanism for the release of biological amine (i.e., dopamine, adrenaline, noradrenaline, serotonin, histamine). These chemical compounds perform a wide range of functions in living organisms [5,6,7,8,9,10].

Amperometry is arguably the most widely used technique for studying the exocytosis of biological amines. Wightman and collaborators introduced single-cell amperometry recordings with carbon fiber by placing the electrode onto the cell membrane, creating an ‘artificial synapse’ [11,12,13,14,15]. This electrochemical technique applies an electrical potential to the electrode surface to oxidize the amines released by the cell, allowing the monitoring of single exocytotic events with superb time resolution.

Platelets are the easiest human cells to study the amines’ exocytosis. They do not synthesize serotonin but avidly uptake it from blood; therefore, over 90% of the blood serotonin is in platelets. Serotonin is mainly stored and released through exocytosis by the δ-granules [16,17]. Amperometry has been applied for monitoring serotonin exocytosis in platelets, including humans, notably by the group of Haynes using single-cell recordings with conventional glass-encapsulated carbon fiber amperometry [18,19,20,21]. However, a given platelet contains just 4–8 δ-granules, which can be released on the opposite side from the electrode, making the process extremely tedious, especially for diagnostic purposes using human platelets.

The usage of conventional multi-electrode array (MEA) devices has significantly streamlined amperometry, eliminating the need for costly equipment such as amplifiers, micromanipulators, drug delivery puffers, antivibration tables, large Faraday cages, or inverted microscopes. Furthermore, these devices require minimal bench space. Most importantly, they enable simultaneous recordings from multiple working electrodes. MEA devices address challenges associated with cell-to-cell recordings, a particularly critical aspect for platelets given their smaller size compared to conventional glass or plastic-encapsulated electrodes.

Recently, our research group measured the release of serotonin by exocytosis in human platelets with boron-doped diamond (BDD) on silicon multi-electrode arrays (BDD-on-silicon MEAs), obtaining similar results to those described by Haynes et al. [17]. MEA devices allow the parallel monitoring of amperometric current from several electrodes simultaneously, increasing the chances of detecting exocytotic signals. Moreover, platelets do not need to be cultured on the device; instead, they are dispersed onto the MEA in a solution and allowed to settle down for a few seconds before reaching the electrodes.

MEA devices for amperometric measurements have been developed using different materials, such as those based on synthetic nanocrystalline BDD microelectrodes grown by chemical vapor deposition (CVD), or more recently, MEAs fabricated within an artificial single-crystal diamond matrix [22,23,24,25,26,27].

This later MEA fabrication utilizes an advanced ion beam lithography technique [28,29] with energy in the order of megaelectronvolts (MeVs), which created 3D micrographitic tracks consisting of buried channels ending with surface electrodes [27,30,31,32,33].

Despite the advantages of the devices used for platelets so far, they are opaque, hindering simultaneous observations of platelets. Given the need for the careful handling of platelets, and considering that certain treatments may promote their aggregation, it becomes crucial to exert control over their physical distribution. In response to this challenge, we chose to implement a novel MEA device that combines the excellent electrochemical characteristics of BDD-on-silicon with transparency, allowing for simultaneous observations. These devices will be employed for fluorescence microscopy.

In this research article, we compare two already described MEA devices: the BDD-on-glass MEA [27] and the BDD-on-silicon MEA [17], with a novel transparent MEA device called the BDD-on-quartz MEA. We describe their general electrochemical characteristics and their ability for amperometric monitoring of serotonin exocytosis from human platelets.

## 2. Materials and Methods

### 2.1. Solutions Used, Unless Specified (in mM), pH Adjusted with NaOH

Phosphate-buffered saline (PBS): NaCl (154), KH_2_PO_4_ (1.08), Na_2_HPO_4_ (5), and pH 7.4.

HEP buffer: NaCl (140), KCl (2.7), ethylene glycol-bis (β-aminoethyl ether)-N,N,N′,N′-tetraacetic acid (EGTA) (5), N-2-Hydroxyethylpiperazine-N′-2-Ethanesulfonic Acid (HEPES) (3.8), penicillin (100 U/mL), gentamicin (40 µg/mL), and pH 7.4.

Citrate buffer: NaCl (150), EDTA (1), glucose (50), Na^+^-citrate (10), penicillin (100 U/mL), gentamicin (40 µg/mL), and pH 7.4.

Additive solutions for platelet conservation:

Buffer 1: NaCl (69.3), KCl (5), MgCl_2_ (1.5), Na_2_HPO_4_/NaH_2_PO_4_ (28.2), Na^+^-citrate (10.8), Na^+^-acetate (32.5), glucose (5), and pH 7.2.

Buffer 2: NaCl (69.3), KCl (5), MgCl_2_ (1.5), Na_2_HPO_4_/NaH_2_PO_4_ (28.2), Na^+^-citrate (10.8), Na^+^-acetate (32.5), glucose (5), ascorbic acid (10 µM), prostaglandin E_1_ (1 µM), penicillin (100 U/mL), gentamicin (40 µg/mL), and pH 7.2.

Thrombin solution: thrombin 4 UI; the final concentration diluted in buffer 1.

### 2.2. Human Platelets Preparation

Human platelet isolation was conducted by modifying the Abcam^®^ method (Cambridge, UK) [16,17]. Briefly, nine milliliters of blood were taken by venipuncture from 10 participating volunteers. This study was approved by the Ethical Committee of the Canary Islands Health Department (Protocol CHUC_2020_80) and by the Ethical Committees of the University Hospital and the University of La Laguna (Protocol CEIBA2020-0430).

Blood samples were subjected to serial centrifugation at room temperature with no brake [17]: (i) 200× *g* for 20 min; separating two-thirds of the supernatant or PRP (platelet-rich plasma); (ii) the PRP was mixed in a 1:1 ratio with a solution of HEPES buffer supplemented with prostaglandin E_1_ (1 µM, final concentration). The mixture was centrifuged at 100× *g* for 15 min; (iii) the supernatant resulting from this second centrifugation was transferred to a sterile tube and centrifuged at 800× *g* for 20 min. The supernatant was discarded by decantation, and the platelet pellet was washed twice with 1 mL of citrate buffer solution. (iv) This pellet was resuspended in 5 mL of buffer 2. The final density of the platelet suspension was adjusted to 10^5^/µL with buffer 2 and was tested by turbidimetry and hemocytometer counting [17]. 

The platelet solution was maintained in bioreactor tubes at room temperature in a humidified environment. To avoid platelet aggregation and activation, the samples described were rotated at 30 rpm at an angle of 30° throughout the conservation time. In a set of previous experiments, we determined that platelets maintain full functionality for 3 days. Nevertheless, all results presented in this paper were obtained in 24–48 h from extraction. The concentration of platelets used in the amperometric measurements was 4 × 10^5^/µL. The discarded samples were disinfected with a 20% bleach solution for 20 min and placed in biological waste buckets for subsequent collection by accredited companies.

### 2.3. BDD-on-Quartz MEA

**Figure 1:**
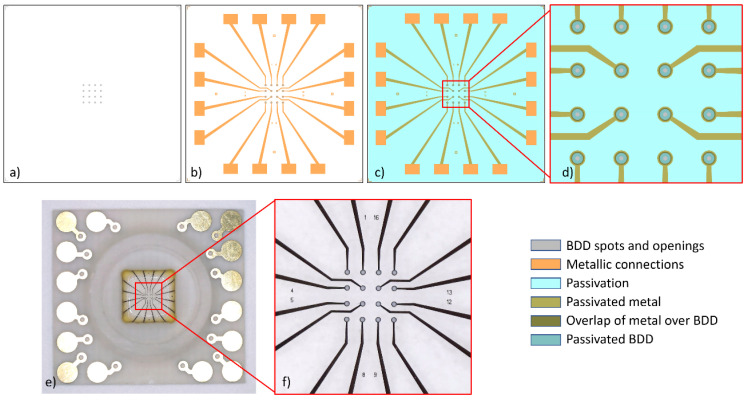
**The BDD-on-quartz MEA.** Layers sequence of the MEA fabrication: (**a**) BDD, (**b**) metallic connections, (**c**) passivation, (**d**) magnification of the layout core region (explanation in text). (**e**) Upper view of the finished device assembled on the carrier board with the glass ring. (**f**) Magnified view of the core region with the µ-electrodes, showing the central transparent region of the BDD spots. The circular active area of the microelectrodes is 20 µm in diameter. The chip size is 6 × 6 mm^2^, and the chamber allows adding 50–200 µL of platelet suspension.

The main reason for developing a new technology for transparent MEAs resides in the fact that as reported in a very comprehensive analysis [34], BDD-on-glass materials exhibit a noise level that is too high for recording exocytosis from platelets. Other secretory biological models, like chromaffin cells and rat pheochromocytoma PC12 cells, provide amperometric spikes of several tens and in some cases even a few hundreds of picoamperes. In the case of platelets, the amperometric spikes can hardly reach an intensity of 10 pA. BDD-on-silicon MEAs were demonstrated to be suitable for these challenging recordings; therefore, we aimed to achieve similar results with a new transparent device: the BDD-on-quartz MEA. However, this new technology is required to face new challenges due to the large mismatch of the thermal expansion between diamond and quartz (see Figure S1 in the Supplementary Material). Both materials are very rigid, and after growing the diamond at about 800 °C, when cooling down to room temperature, the diamond film is affected by a significant tensile stress. If the diamond film is very thin, up to a few tens of nanometers, there is enough elasticity for dampening such stress, but if the grown layer is a few microns thick, the diamond film cracks in a myriad of fragments, like a mosaic. The solution to this problem could be found by observing that the size of such fragments ranges between 100 and 500 µm (see Figure S2 in the Supplementary Material). On this basis, it appeared realistic to selectively grow the diamond only for the electrode structures, for which we decided to us round spots of 60 µm in diameter, forming a planar 4 × 4 array with a pitch of 200 μm. Then, the spots are connected to the bonding pads and placed at the edge of the chip by means of planar metal wires. The contact resistance with the BDD spots is minimized by adopting ring-shaped metal structures, which leave a transparent central region with a diameter of 45 µm (Figure 1). 

**Figure 2:**
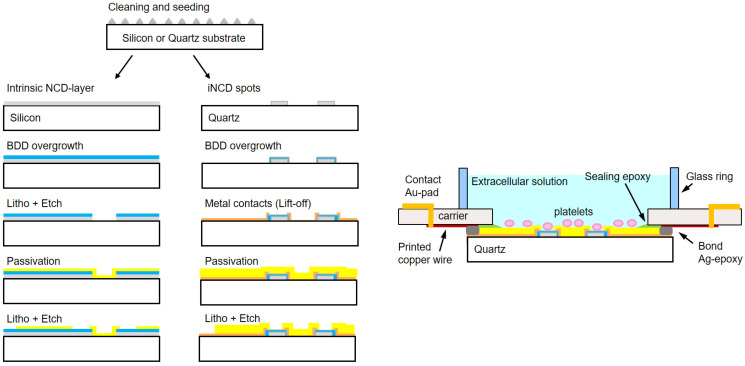
**Fabrication of MEAs.** (**Left**) Schematic comparison of the fabrication steps for the two different technologies, i.e., the BDD-on-silicon MEA and the BDD-on-quartz MEA. (**Right**) Scheme of the assembled BDD-on-quartz MEA.

The fabrication is illustrated in the top-view in Figure 1 and the technological protocol in Figure 2. It consists schematically of the following steps:

1. Clean and then spin-coat the wafer with a NanoAmando seeding solution (New Metals and Chemicals, Tokyo, Japan).
2. Grow a 50 nm thin intrinsic nanocrystalline diamond (iNCD) layer by microwave plasma chemical vapor deposition (MWCVD) at a power of 2200 W in an H_2_ atmosphere at a gas flow of 400 sccm, with 1.5% of CH_4_, at a temperature of 800 °C and pressure of 30 Torr. This growth step has a duration of 10 min.
3. Create the wafer by lifting off a titanium hard mask with a pattern of the 60 µm spots.
4. Etch the unprotected iNCD by reactive ion etching (RIE) in an Ar-O_2_ atmosphere. This leaves the ‘footprint’ for growing the diamond spots of the microelectrodes.
5. Grow the iNCD spots resulting from the previous step up to a thickness of 1 µm. This growth is carried out with the same modality of step 2 but for a duration of 190 min.
6. Overgrow the iNCD-spots with ~350 nm BDD in a doping-dedicated MWCVD-reactor. Parameters are the same as above for steps 2 and 5, but the process duration is 70 min in this case. Doping is provided by boron wires (Goodfellow, Bad Nauheim, Germany) introduced into the plasma.
7. Create the wafer by lifting off the metal ring contacts and wires out of 100 nm titanium and 50 nm gold.
8. Passivate the wafer with a polyimide-based photoresist (Durimide^®^ 7505, Fujifilm, Tokyo, Japan) and transfer the pattern of the openings of electrodes and contact pads by lithography.
9. Dice the wafer, bind the chips onto appropriate polychlorinated biphenyl carriers (PCB carriers), and glue a 10 mm wide and 4 mm thick glass ring to provide a ~200 µL incubation volume.

In summary, BDD-on-quartz MEAs have the same functional layout and dimensions as BDD-on-silicon MEAs, with 16 BDD microelectrodes (each with a nominal diameter of 20 µm) placed on a 4 × 4 array with a pitch of 200 µm to avoid overlapping the signals recorded individually by each microelectrode. 

The comparison of these two technologies regards primarily the materials, with their specific processes adopted for the various structures, as summarized in Table 1 below:

### 2.4. BDD-on-Quartz MEA Recording System

Amperometric measurements of serotonin release by exocytosis were carried out with the same system previously used by our research group for this type of measurement with BDD-on-silicon MEA devices; see González-Brito et al., 2023, for more details [17]. Briefly, the read-out electronics consists of 16 transimpedance amplifiers with 1 GΩ feedback resistance, followed by anti-aliasing Bessel low-pass filters of the 4th order with a cutoff at 1 kHz. Then, the signals are acquired at a sampling rate of 4 kHz per channel and a 16-bit resolution with a USB-6216 from National Instruments (NI, Austin, TX, USA), controlled with an appropriate LabVIEW program, and are stored on the hard disk of the PC. Electrodes were calibrated with norepinephrine solutions (Figure S7 Supplementary Materials).

Exocytotic events are detected as oxidation currents by biasing the BDD electrodes at +800 mV against an Ag/AgCl sintered pellet electrode immersed in an incubation bath. Considering that this electrode is non-polarizable and that its effective active area of a few hundred mm^2^ is about five orders of magnitude larger than the total active area of the 16 electrodes together, providing a very low contact resistance to the electrolyte, the functions of a reference electrode and a counter electrode can be merged in this single electrode without introducing relevant systematic errors. This electrochemical scheme is very commonly adopted in cellular electrophysiology.

BDD-on-quartz MEA chips were used in each individual measurement. The goal was to demonstrate that these new transparent MEAs allow us to carry out amperometric measurements with the same efficiency and sensitivity as the opaque and easier-to-fabricate BDD-on-silicon MEA devices while adding transparency to allow optical inspections and detection methods.

### 2.5. Amperometric Data Analysis

Data analysis and graphing were carried out using IGOR-Pro 8 (Wavemetrics, Portland, OR, USA). Macros and routines were a modification of our previous version [35] to allow automatic data management. The macros were designed to obtain the following kinetic data from the recorded signals: Imax, maximum oxidation current, expressed in pA; t_1/2_, spike full width at half maximum (FWHM), expressed in ms; Q, spike net charge, expressed in pC; and m, ascending slope of the spike, expressed in pA/ms. These macros are freely available upon request. The mean values of the kinetic parameters are presented. 

The discrimination threshold was fixed at 2.5 standard deviations (SDs) of the basal noise of the first derivative of each recording. It usually includes spikes with an Imax larger than 1.5–2 pA. All those spikes passed the selection criteria: (1) spikes above the detection threshold, (2) without overlap, and (3) measured parameters not affected by any artifact during recording.

### 2.6. Microscopy Observation

**Figure 3:**
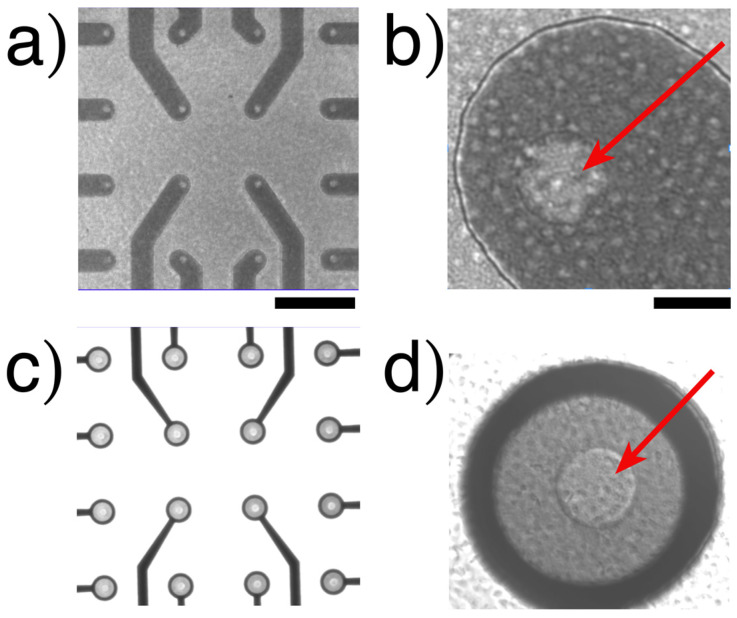
**Platelets on a transparent MEA device.** (**a**) A low magnification of the 16 electrodes of BDD on a glass carrier. (**b**) A high magnification of an electrode. (**c**,**d**) are from BDD on quartz. The only active surfaces are the inner circle (arrows). Notice the presence of platelets at higher magnifications. Calibration bar (**a**,**c**) = 200 µm; (**b**,**d**) = 20 µm. No images can be obtained through opaque silicon chips (see more images in Figures S3 and S4 in the Supplementary Material).

The images shown in Figure 3 (only on transparent MEAs) were obtained by differential interference contrast (DIC) microscopy. We use observations as a qualitative control for platelet density, assuring that platelets obtain all electrodes without forming aggregate lumps, assuming identical cell distribution on the BDD-on-silicon MEA.

The microscopy images depict a homogeneous distribution of the sample in each BDD-on-quartz MEA device (refer to Figures S3 and S4 in the Supplementary Material). For each measurement, 133 µL of the PRP sample was added at a concentration of 4 × 10^5^/µL (approximately 53 million platelets per experiment). Each device features 16 microelectrodes, each with a nominal diameter of 20 µm and a surface area of ≈314 µm^2^. The total active surface of the device is ≈5000 µm^2^. This provides a sufficient number of cells on the electrodes for amperometric recordings.

### 2.7. Statistics

All kinetic parameters are presented as means ± SEM (the standard error of the mean). The non-parametric Mann–Whitney rank sum test or Student’s *t*-test were the mathematical parameters used to evaluate the statistical significance between groups of experiments (as appropriate, based on the D’Agostino–Pearson normality test). Differences were considered significant at the indicated *p* level. Data analysis was conducted using Prism^®^ Software 5.03 (GraphPad Software, San Diego, CA, USA).

## 3. Results

### 3.1. Characterization and Comparison of BDD-on-Quartz MEAs and BDD-on-Silicon MEAs

The MEAs were initially characterized using cyclic voltammetry (CV) in PBS, conducting multiple scans to assess electrode activity and stability cycle after cycle. The performances were nearly identical (Figures S5 and S6, Supplementary Material) in terms of potential window, peak currents, and background current.

Subsequently, the background noise of the MEAs was analyzed by recording signals without cells, covering a bandwidth of 1 kHz at a sampling rate of 4 kHz per channel, and performing a Fast Fourier Transform (FFT) to obtain noise spectra. Figure S6 in the Supplemental Material demonstrates very similar performances in terms of average noise spectral density. However, the spread of the individual spectra is lower for the BDD-on-quartz MEA. This result may be attributed to the lower resistance of the metal connections compared to the BDD connections on the BDD-on-silicon MEA. This leads to a more uniform noise level because the dominant noise sources are the microelectrodes on the BDD spots, which are substantially identical. The overall performance of BDD-on-glass is poorer, in particular regarding the noise level, which is one order of magnitude larger (Figures S5 and S6, Supplementary Material).

Microelectrode calibration was conducted using incremental concentrations of norepinephrine in buffer 1. The final calibration concentrations were 0, 1.25, 2.5, 5, and 10 µM. This method revealed that the variation in response among the 16 microelectrodes within the same device was less than 10% (refer to Figure S7 in the Supplementary Material). The calibration experiment confirmed the stable performance of the microelectrodes and their capability for accurate quantitative measurements.

### 3.2. Characterization of Serotonin Exocytosis of Human Platelets Using the BDD-on-Quartz MEA

**Figure 4:**
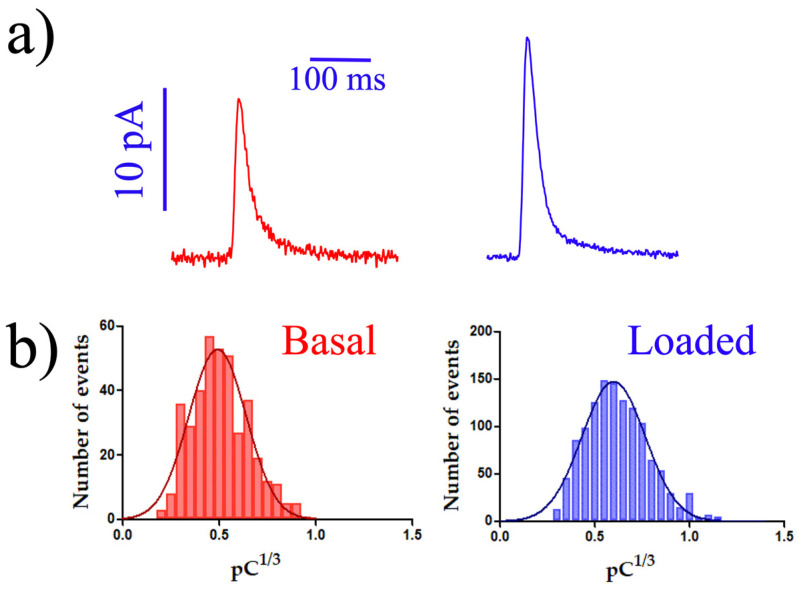
**Amperometric recordings using the BDD-on-quartz MEA.** (**a**) Representative secretory spikes depicted with the values obtained under basal conditions (red spike) and after loading with 10 µM serotonin for 2 h (blue spike). For this representation, spikes were constructed using the average values in Table 2. (**b**) Histogram distribution using all the spikes obtained in each condition: in the absence (red bars) and after a 2 h of incubation with 10 µM serotonin (blue bars). n = 421 and 1230 spikes from basal and loaded platelets, respectively.

Table 2 displays the main values of the four major kinetic parameters of secretory spikes, validating the results obtained with the BDD-on-quartz MEA. In general, all data exhibited good detection capacity and reproduced those obtained with the BDD-on-silicon MEA [17] and single electrodes of encapsulated carbon fiber electrodes [21]. Nevertheless, both the BDD-on-silicon and BDD-on-quartz MEA exhibited better signal-to-noise ratios. Additionally, both are easily and robustly cleaned and reused. Figure 4a shows representative spikes constructed using the average values in Table 2.

This set of information validates the use of these transparent devices for conducting amperometric measurements of electroactive molecules released by cells through secretory pathways. The results obtained are summarized in the statistically calculated mean peaks and the histograms presented in Figure 4. The histograms represent the Q^1/3^, or cubic root of the net charge (Q) of the recorded peaks, allowing the representation and analysis of the quantum size distribution of secretion vesicles released through exocytosis by the human platelets studied (because the sphere volume is proportional to the third power of its radius) [36,37,38,39].

All studies were conducted on isolated human platelets, closely maintained under physiological conditions following the general guidelines from clinical blood banks. We verified that amperometric measurements with the BDD-on-silicon MEA were effective both under basal conditions and in serotonin-loaded conditions.

Platelets avidly uptake serotonin through specific mechanisms, including SERT-1, on their membranes and VMAT-2 on dense granules. Additionally, serotonin permeates through a concentration gradient and accumulates within the acidic dense granules. In amperometry experiments, we assess both the baseline serotonin content and the functional condition of granule uptake. The incubation with 10 µM serotonin for 2 h was established in previous experiments [16] and is now employed as a control for loading.

## 4. Discussion 

PRP has proven to be an excellent source of platelets for conducting amperometric studies of serotonin released through exocytosis. These investigations served as a foundational step to validate the efficacy of the BDD-on-silicon MEA under both basal conditions and after loading human platelets with 10 µM serotonin for 2 h. We established these incubation conditions in a previous paper [17]. Advancing further, we have developed a new BDD microelectrode device on a quartz carrier. The physicochemical nature of this support enables the passage of various light wavelengths, rendering the new device, termed the BDD-on-quartz MEA, transparent and suitable for certain microscopic studies.

Similar to the BDD-on-silicon MEA, the BDD-on-quartz MEA enables the recording of amperometry in human platelets with an excellent signal-to-noise ratio (Figure S6 in the Supplementary Material). With these two devices, we can presently measure spikes as small as 1.5–1.8 pA. Consequently, we can conduct a quantitative study of serotonin release in platelets under both basal conditions and after loading with 10 µM serotonin for 2 h (Figure 4). The kinetic parameters obtained with BDD-on-quartz MEAs are indistinguishable from those acquired with conventional amperometry [21] or with the BDD-on-silicon MEA system [17]. While secretory spikes generally occur spontaneously, the kinetics of exocytotic events remain almost identical, even when induced by the addition of thrombin.

Selectivity becomes a challenge when dealing with various detectable molecules exhibiting similar behavior, such as oxidation potential and the time course of the reaction. In our study, our focus is solely on serotonin, the exclusive electrochemically active molecule released through platelet exocytosis. Even if other oxidizable species are present, possibly as contaminations in the incubation chamber, they would not produce exocytosis-like signal transients. Instead, they would result in nearly constant currents (quasi-DC) over extended time intervals. Therefore, selectivity is inherently provided by the cell model and the preparation of the cell suspension. 

### 4.1. Different Types of Recorded Spikes

**Figure 5:**
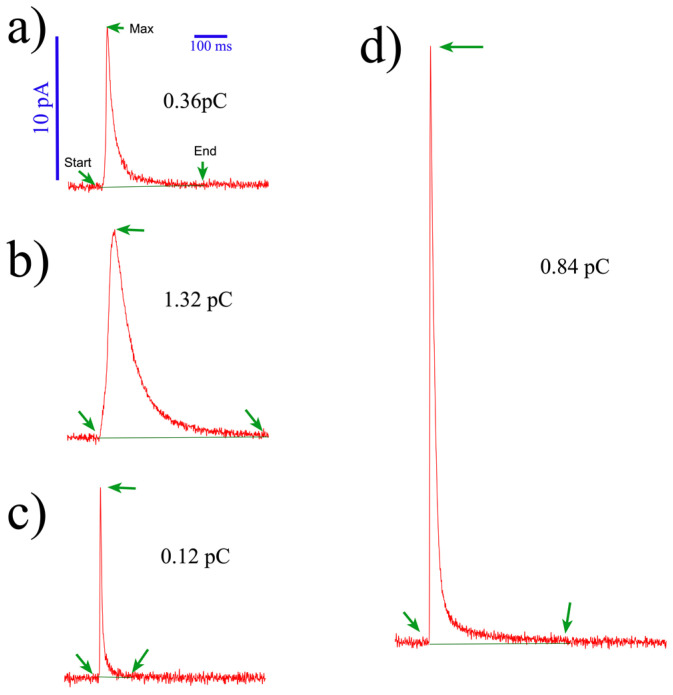
**Heterogeneity of secretory spikes from human platelets.** The figure shows some amperometric spikes obtained from a single experiment. Each trace corresponds to the serotonin release single event exocytosis recorded with BDD-on-quartz MEA devices. Notice the different shapes and kinetics (**a**–**d**). All spikes are on the same scale. Notice the different kinetics and quantum sizes, expressed in pC. Spike characterization was carried out using our macros for IgorPro 8^®^ [35]. These macros can be freely shared upon request.

Observing the recorded signals from human platelets reveals a high heterogeneity in the kinetics and quantum size of amperometric spikes (Figure 5). This phenomenon is well documented and common to other cell types [40,41,42,43], such as chromaffin [44,45], PC12 [46,47], or mast cells, which have been associated with different types of exocytosis (full vs. partial release) [47]. Factors such as intravesicular content and its inner distribution (matrix vs. halo) [47], fusion pore kinetics, and its expansion [48,49] contribute to this heterogeneity. Additionally, biological factors, electrode sensitivity, the distance from the electrode to the exocytotic site, and any factor modifying the electrode performance [35] can impact amine detection. Researchers should be aware of the varying sensitivities of electrodes within a chip and between different chips. Thus, ensuring electrode responsiveness through cyclic voltammetry performance (Figure S5 in the Supplementary Material) and pre- and post-calibrations with proper standards is crucial.

However, employing simple average measurements for the comparison of two experimental conditions should be approached with caution. For instance, ensuring similarity in the number of spikes is crucial; using ‘N’ instead of ‘n’ (representing the number of individuals versus the number of spikes) introduces less bias. Since the spike distribution is not normal, statistical comparisons are often conducted using medians instead of means.

### 4.2. Brief Comparison of the Three BDD MEAs

#### 4.2.1. BDD-on-Glass MEAs

Devices with a boron silicate matrix produce deficient amperometric recordings with human platelets to date. As mentioned earlier, the background noise is excessively high for platelet recordings. While their fabrication technology is simpler than that of the other two MEA types, making these chips appealing for mass production at a lower cost, their microelectrodes are not stable during the cleaning process. They frequently become partially detached and lost. Currently, we only use these transparent devices as a parallel control for platelet allocation on the chip surface because their electrode distribution is the same as that of opaque silicon.

#### 4.2.2. BDD-on-Silicon MEAs

These devices allow amperometric, potentiometric, and voltammetric measurements. Another advantage is the production cost compared to other existing MEA devices with a single crystal diamond (for example, micrographitic–diamond–multi-electrode arrays (μG-D-MEAs)) [26,27,33]. BDD-on-silicon MEAs are very low-noise devices, stable, and easy to clean. As platelets are not cultured on these chips but acutely added, biocompatibility is not a real problem. In our hands, these devices are ideal for the amperometric measurements of serotonin exocytosis in human platelets [17].

#### 4.2.3. BDD-on-Quartz MEAs

These devices maintain a similar structural arrangement as the BDD-on-silicon MEA and are transparent, allowing cellular imaging studies to be carried out (Figure 3). However, platelets are too small for observing single exocytotic occurrences, and our efforts to observe coincident events using quinacrine or acridine orange were not yet successful.

BDD-on-quartz MEAs, like BDD-on-silicon MEAs, have been shown to be effective in amperometric measurements of serotonin release through exocytosis in human platelets. Amperometrical recordings are comparable. The main limitation of BDD on quartz surfaces is the more complex fabrication protocol, which is necessary for dealing with the thermal expansion mismatch between BDD and the quartz carrier.

## 5. Conclusions

The results presented in this paper confirm the effectiveness of the new BDD-on-quartz MEA devices in studying and quantifying the release of serotonin from human platelets. While this study was initially intended for working with human cells, we are currently expanding its capabilities to record catecholamine exocytosis from chromaffin and PC12 cells. Furthermore, the simultaneous observation of exocytosis would allow for the characterization of the different modes of exocytotic release [50].

Regarding the sensitivity and reproducibility of amperometric responses, the overall characteristics of the BDD-on-quartz MEA are nearly identical to the BDD-on-silicon MEA [11]. The BDD-on-quartz MEA also offers the possibility of combining amperometry with microscopy. Since platelets are the most accessible human source of amine-containing cells and are frequently used as a model for neurological diseases [51,52,53,54,55,56], MEA devices show promise as tools for translational medicine [57,58] and as a method to characterize human platelets for their potential clinical uses [59,60,61,62].

## Figures and Tables

**Table 1:** Comparison of the structural materials adopted for the three MEA technologies.

MEA Material	BDD-on-Glass	BDD-on-Silicon	BDD-on-Quartz
Substrate wafer	AF32Eco ^1^	Silicon	Amorphous quartz
Microelectrodes	BDD	BDD	BDD
Connecting wires	BDD	BDD	Titanium/Gold
Passivation	Stack of SiO/SiN	Stack of SiO/SiN	Polyimide
Bonds	Silver-epoxy	Silver-epoxy	Silver-epoxy
Carrier	PCB	PCB	PCB

^1^ Alkali-free alumina–borosilicate thin glass.

**Table 2:** Kinetic parameters of amperometric spikes using the BDD-on-quartz MEA. Data from 10 human subjects. Data are expressed as means ± SEM. Calculations were performed on the N basis (the averages are calculated taking the peaks of each subject, and the results are used to obtain the statistical values). These values are used for drawing the spikes shown in Figure 4.

MEA	Imax (pA)	Q (pC)	t_1/2_ (ms)	m (nA/s)
**Basal**	6.59 ± 0.35	0.17 ± 0.02	17.3 ± 1.25	1.43 ± 0.07
**5-HT loaded**	9.70 ± 0.71 **	0.30 ± 0.02 ***	28.9 ± 1.22 **	1.76 ± 0.14 ^n.s.^

** *p* < 0.005; *** *p* < 0.001. ^n.s.^: not significant. Student’s *t*-test.

## Data Availability

The data presented in this study are available in the Supplementary Materials.

## Supplementary Materials

The following supporting information can be downloaded at https://www.mdpi.com/article/10.3390/bios14020075/s1, Figure S1. SM—Linear expansion over the temperature of silicon, diamond, and quartz, showing the expansion mismatch and the related tensile stress when cooling down after CVD growth. Figure S2. SM—Fragmentation of a 4 µm thick diamond-on-quartz layer: most fragments have a size between 100 and 500 µm. Figure S3. SM—Images of platelets on the BDD on the quartz device. Figure S4. SM—Images of platelets distributed homogeneously in buffer 1 over microelectrodes and nearby on the BDD-on-quartz and BDD-on-glass devices. Figure S5. SM—Data of cyclic voltammetry (CV) in PBS. Comparison of CV plots recorded at a scan rate of 200 mV/s. Figure S6. SM—Noise spectra. Figure S7. SM—Calibration lines of the BDD-on-quartz MEA device.

## References

[1] Wightman R.M., Haynes C.L. (2004). Synaptic vesicles really do kiss and run. Nat. Neurosci..

[2] Li X., Majdi S., Dunevall J., Fathali H., Ewing A.G. (2015). Quantitative Measurement of Transmitters in Individual Vesicles in the Cytoplasm of Single Cells with Nanotip Electrodes. Angew. Chem. Int. Ed. Engl..

[3] Ren L., Mellander L.J., Keighron J., Cans A.-S., Kurczy M.E., Svir I., Oleinick A., Amatore C., Ewing A.G. (2016). The evidence for open and closed exocytosis as the primary release mechanism. Q. Rev. Biophys..

[4] Wang Y., Ewing A. (2021). Electrochemical Quantification of Neurotransmitters in Single Live Cell Vesicles Shows Exocytosis is Predominantly Partial. ChemBioChem.

[5] Carlsson A. (1987). Perspectives on the Discovery of Central Monoaminergic Neurotransmission. Annu. Rev. Neurosci..

[6] Greengard P. (2001). The Neurobiology of Slow Synaptic Transmission. Science.

[7] Engle K., Zhou M., Wang J. (2004). Identification and Characterization of a Novel Monoamine Transporter in the Human Brain. J. Biol. Chem..

[8] Capellino S., Claus M., Watzl C. (2020). Regulation of natural killer cell activity by glucocorticoids, serotonin, dopamine, and epinephrine. Cell. Mol. Immunol..

[9] Jiang Y., Zou D., Li Y., Gu S., Dong J., Ma X., Xu S., Wang F., Huang J.H. (2022). Monoamine Neurotransmitters Control Basic Emotions and Affect Major Depressive Disorders. Pharmaceuticals.

[10] Moura C., Vale N. (2023). The Role of Dopamine in Repurposing Drugs for Oncology. Biomedicines.

[11] Leszczyszyn D.J., Jankowski J.A., Viveros O.H., Diliberto E.J., Near J.A., Wightman R.M. (1990). Nicotinic receptor-mediated catecholamine secretion from individual chromaffin cells. Chemical evidence for exocytosis. J. Biol. Chem..

[12] Leszczyszyn D.J., Jankowski J.A., Viveros O.H., Diliberto E.J., Near J.A., Wightman R.M. (1991). Secretion of catecholamines from individual adrenal medullary chromaffin cells. J. Neurochem..

[13] Wightman R.M., Jankowski J.A., Kennedy R.T., Kawagoe K.T., Schroeder T.J., Leszczyszyn D.J., Near J.A., Diliberto E.J., Viveros O.H. (1991). Temporally resolved catecholamine spikes correspond to single vesicle release from individual chromaffin cells. Proc. Natl. Acad. Sci. USA.

[14] Lemaître F., Collignon M.G., Amatore C. (2014). Recent advances in Electrochemical Detection of Exocytosis. Electrochim. Acta.

[15] Li Y.-T., Zhang S.-H., Wang L., Xiao R.-R., Liu W., Zhang X.-W., Zhou Z., Amatore C., Huang W.-H. (2014). Nanoelectrode for Amperometric Monitoring of Individual Vesicular Exocytosis Inside Single Synapses. Angew. Chemie Int. Ed..

[16] Montenegro P., Pueyo M., Lorenzo J.N., Villar-Martinez M.D., Alayón A., Carrillo F., Borges R. (2020). A Secretory Vesicle Failure in Parkinson’s Disease Occurs in Human Platelets. Ann. Neurol..

[17] Brito R.G., Montenegro P., Méndez A., Carabelli V., Tomagra G., Shabgahi R.E., Pasquarelli A., Borges R. (2023). Multielectrode Arrays as a Means to Study Exocytosis in Human Platelets. Biosensors.

[18] Ge S., Wittenberg N.J., Haynes C.L. (2008). Quantitative and Real-Time Detection of Secretion of Chemical Messengers from Individual Platelets. Biochemistry.

[19] Ge S., White J.G., Haynes C.L. (2009). Quantal Release of Serotonin from Platelets. Anal. Chem..

[20] Ge S., Woo E., Haynes C.L. (2011). Quantal Regulation and Exocytosis of Platelet Dense-Body Granules. Biophys. J..

[21] Ge S., Woo E., White J.G., Haynes C.L. (2011). Electrochemical measurement of endogenous serotonin release from human blood platelets. Anal. Chem..

[22] Gao Z., Carabelli V., Carbone E., Colombo E., Dipalo M., Manfredotti C., Pasquarelli A., Feneberg M., Thonke K., Vittone E. (2011). Transparent microelectrode array in diamond technology. J. Micro-Nano Mech..

[23] Kiran R., Rousseau L., Lissorgues G., Scorsone E., Bongrain A., Yvert B., Picaud S., Mailley P., Bergonzo P. (2012). Multichannel Boron Doped Nanocrystalline Diamond Ultramicroelectrode Arrays: Design, Fabrication and Characterization. Sensors.

[24] Gillis K.D., Liu X.A., Marcantoni A., Carabelli V. (2018). Electrochemical measurement of quantal exocytosis using microchips. Pflugers Arch..

[25] Purcell E.K., Becker M.F., Guo Y., Hara S.A., Ludwig K.A., McKinney C.J., Monroe E.M., Rechenberg R., Rusinek C.A., Saxena A. (2021). Next-Generation Diamond Electrodes for Neurochemical Sensing: Challenges and Opportunities. Micromachines.

[26] Picollo F., Battiato A., Carbone E., Croin L., Enrico E., Forneris J., Gosso S., Olivero P., Pasquarelli A., Carabelli V. (2015). Development and Characterization of a Diamond-Insulated Graphitic Multi Electrode Array Realized with Ion Beam Lithography. Sensors.

[27] Granado T.C., Neusser G., Kranz C., Filho J.B.D., Carabelli V., Carbone E., Pasquarelli A. (2015). Progress in transparent diamond microelectrode arrays. Phys. Status Solidi A.

[28] Watt F., Bettiol A.A., Van Kan J.A., Teo E.J., Breese M.B.H. (2005). Ion Beam Lithography and Nanofabrication: A Review. Int. J. Nanosci..

[29] Sharma E., Rathi R., Misharwal J., Sinhmar B., Kumari S., Dalal J., Kumar A. (2022). Evolution in Lithography Techniques: Microlithography to Nanolithography. Nanomaterials.

[30] Picollo F., Battiato A., Bernardi E., Boarino L., Enrico E., Forneris J., Gatto Monticone D., Olivero P. (2015). Realization of a diamond based high density multi electrode array by means of deep ion beam lithography. Nucl. Instrum. Methods Phys. Res. Sect. B Beam Interact. Mater. At..

[31] Wigström J., Dunevall J., Najafinobar N., Lovrić J., Wang J., Ewing A.G., Cans A.-S. (2016). Lithographic Microfabrication of a 16-Electrode Array on a Probe Tip for High Spatial Resolution Electrochemical Localization of Exocytosis. Anal. Chem..

[32] Picollo F., Battiato A., Boarino L., Tchernij S.D., Enrico E., Forneris J., Gilardino A., Jakšić M., Sardi F., Skukan N. (2017). Fabrication of monolithic microfluidic channels in diamond with ion beam lithography. Nucl. Instrum. Methods Phys. Res. Sect. B Beam Interact. Mater. At..

[33] Tomagra G., Franchino C., Carbone E., Marcantoni A., Pasquarelli A., Picollo F., Carabelli V., Borges R. (2023). Methodologies for Detecting Quantal Exocytosis in Adrenal Chromaffin Cells through Diamond-Based MEAs. Chromaffin Cells.

[34] Pippione G., Olivero P., Fischer M., Schreck M., Pasquarelli A. (2017). Characterization of CVD heavily B-doped diamond thin films for multi electrode array biosensors. Phys. Status Solidi A.

[35] Segura F., Brioso M.A., Gomez J.F., Machado J.D., Borges R. (2000). Automatic Analysis for Amperometrical Recordings of Exocytosis. J. Neurosci. Methods.

[36] Finnegan J.M., Pihel K., Cahill P.S., Huang L., Zerby S.E., Ewing A.G., Kennedy R.T., Wightman R.M. (1996). Vesicular quantal size measured by amperometry at chromaffin, mast, pheochromocytoma and pancreatic beta-cells. J. Neurochem..

[37] Hochstetler S.E., Puopolo M., Gustincich S., Raviola E., Wightman R.M. (2000). Real-Time Amperometric Measurements of Zeptomole Quantities of Dopamine Released from Neurons. Anal. Chem..

[38] Elhamdani A., Palfrey H.C., Artalejo C.R. (2001). Quantal Size Is Dependent on Stimulation Frequency and Calcium Entry in Calf Chromaffin Cells. Neuron.

[39] Qin N., Chen Z., Xue R. (2022). A two-subpopulation model that reflects heterogeneity of large dense core vesicles in exocytosis. Cell Cycle.

[40] Ramachandran S.B., Gillis K.D. (2018). A matched-filter algorithm to detect amperometric spikes resulting from quantal secretion. J. Neurosci. Methods.

[41] Ramachandran S.B., Gillis K.D. (2019). Estimating amperometric spike parameters resulting from quantal exocytosis using curve fitting seeded by a matched-filter algorithm. J. Neurosci. Methods.

[42] Keighron J.D., Wang Y., Cans A.-S. (2020). Electrochemistry of Single-Vesicle Events. Annu. Rev. Anal. Chem..

[43] Hatamie A., He X., Zhang X.-W., Oomen P.E., Ewing A.G. (2023). Advances in nano/microscale electrochemical sensors and biosensors for analysis of single vesicles, a key nanoscale organelle in cellular communication. Biosens. Bioelectron..

[44] Huang M., Delacruz J.B., Ruelas J.C., Rathore S.S., Lindau M. (2018). Surface-modified CMOS IC electrochemical sensor array targeting single chromaffin cells for highly parallel amperometry measurements. Pflug. Arch.—Eur. J. Physiol..

[45] de Diego A.M.G., Ortega-Cruz D., García A.G. (2020). Disruption of Exocytosis in Sympathoadrenal Chromaffin Cells from Mouse Models of Neurodegenerative Diseases. Int. J. Mol. Sci..

[46] Sombers L.A., Hanchar H.J., Colliver T.L., Wittenberg N., Cans A., Arbault S., Amatore C., Ewing A.G. (2004). The effects of vesicular volume on secretion through the fusion pore in Exocytotic release from PC12 cells. J. Neurosci..

[47] Gu C., Ewing A.G. (2021). Simultaneous detection of vesicular content and exocytotic release with two electrodes in and at a single cell. Chem. Sci..

[48] de Toledo G.Á., Montes M.A., Montenegro P., Borges R. (2018). Phases of the exocytotic fusion pore. FEBS Lett..

[49] Jackson M.B., Hsiao Y.-T., Chang C.-W. (2020). Fusion pore expansion and contraction during catecholamine release from endocrine cells. Biophys. J..

[50] Machado J.D., Segura F., Brioso M.A., Borges R. (2000). Nitric oxide modulates a late step of exocytosis. J. Biol. Chem..

[51] Leiter O., Walker T.L. (2019). Platelets: The missing link between the blood and brain?. Prog. Neurobiol..

[52] Canobbio I. (2019). Blood platelets: Circulating mirrors of neurons?. Res. Pract. Thromb. Haemost..

[53] Izzi B., Tirozzi A., Cerletti C., Donati M.B., de Gaetano G., Hoylaerts M.F., Iacoviello L., Gialluisi A. (2020). Beyond Haemostasis and Thrombosis: Platelets in Depression and Its Co-Morbidities. Int. J. Mol. Sci..

[54] Tirozzi A., Izzi B., Noro F., Marotta A., Gianfagna F., Hoylaerts M.F., Cerletti C., Donati M.B., De Gaetano G., Iacoviello L. (2020). Assessing genetic overlap between platelet parameters and neurodegenerative disorders. Front. Immunol..

[55] Canobbio I., Barbieri S.S. (2022). Are platelets more than a model of brain neurons?. Bleeding Thromb. Vasc. Biol..

[56] Burnouf T., Walker T.L. (2022). The multifaceted role of platelets in mediating brain function. Blood.

[57] Wang Y., Liu S., Wang H., Zhao Y., Zhang X.-D. (2022). Neuron devices: Emerging prospects in neural interfaces and recognition. Microsyst. Nanoeng..

[58] Liu Y., Xu S., Yang Y., Zhang K., He E., Liang W., Luo J., Wu Y., Cai X. (2023). Nanomaterial-based microelectrode arrays for in vitro bidirectional brain–computer interfaces: A review. Microsyst. Nanoeng..

[59] Everts P., Onishi K., Jayaram P., Lana J.F., Mautner K. (2020). Platelet-Rich Plasma: New Performance Understandings and Therapeutic Considerations in 2020. Int. J. Mol. Sci..

[60] Nebie O., Carvalho K., Barro L., Delila L., Faivre E., Renn T.-Y., Chou M.-L., Wu Y.-W., Nyam-Erdene A., Chou S.-Y. (2021). Human platelet lysate biotherapy for traumatic brain injury: Preclinical assessment. Brain.

[61] Nebie O., Buée L., Blum D., Burnouf T. (2022). Can the administration of platelet lysates to the brain help treat neurological disorders?. Cell Mol. Life Sci..

[62] Burnouf T., Chou M.-L., Lundy D.J., Chuang E.-Y., Tseng C.-L., Goubran H. (2023). Expanding applications of allogeneic platelets, platelet lysates, and platelet extracellular vesicles in cell therapy, regenerative medicine, and targeted drug delivery. J. Biomed. Sci..

