# Supplementary material for: Analytical Determination of Serotonin Exocytosis in Human Platelets with BDD-on-Quartz MEA Devices
Source: Biosensors (Basel). 2024 Jan 31;14(2):75. doi: 10.3390/bios14020075 (PMC10886747; doi:10.3390/bios14020075)
Supplement: Supplementary file 1 [file biosensors-14-00075-s001.zip › biosensors-2775209-supplementary.pdf]

biosensors

MDPI

Supplementary Materials

Analytical determination of serotonin exocytosis in human platelets with BDD-on-quartz MEA devices

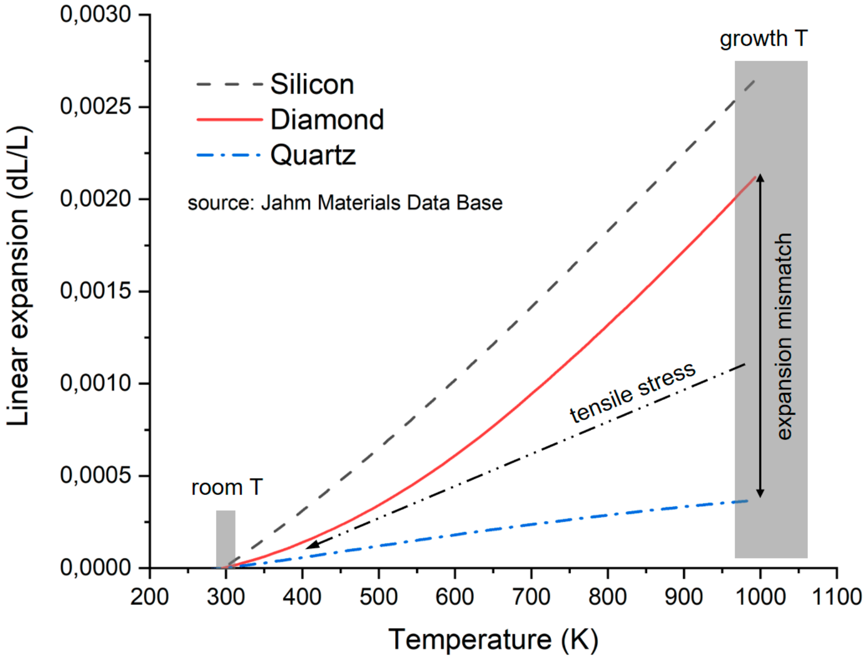

Figure S1. SM—Linear expansion over the temperature of silicon, diamond, and quartz, showing the expansion mismatch and the related tensile stress when cooling down after CVD growth.

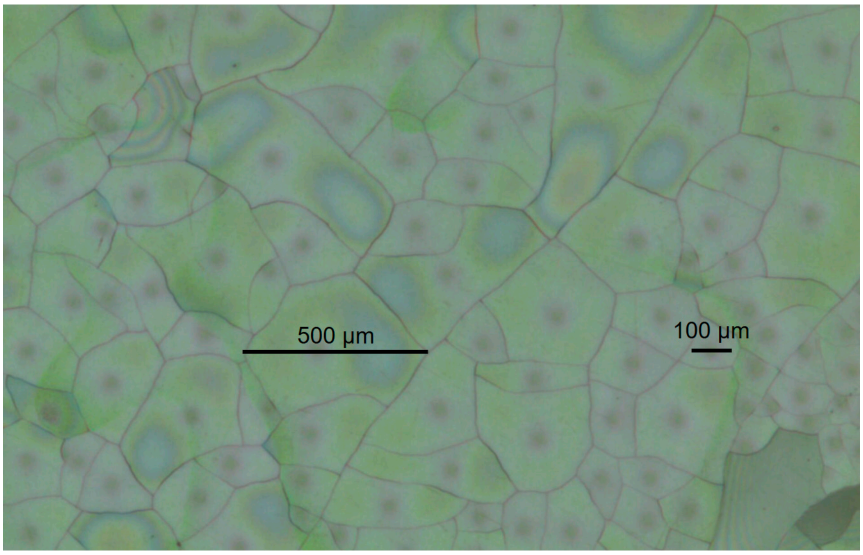

Figure S2. SM—Fragmentation of a 4 µm thick diamond-on-quartz layer: most fragments have a size between 100 and 500 µm.

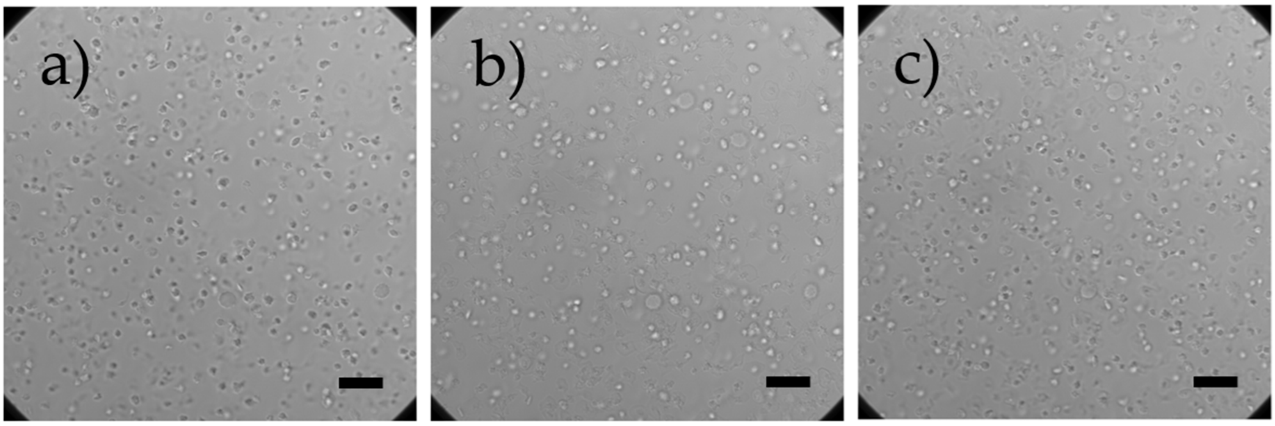

Figure S3. SM—Images of platelets on the BDD-on-quartz device. Platelets can be seen by microscopy on the amorphous quartz surface. Platelets were uniformly distributed in the SSP+ medium used for the measurements. Images were taken at different focal planes of ≈50 μm (**b**) and 100 μm (**c**) from the bottom (**a**), revealing the presence of "floating" platelets. Calibration bars = 20 μm.

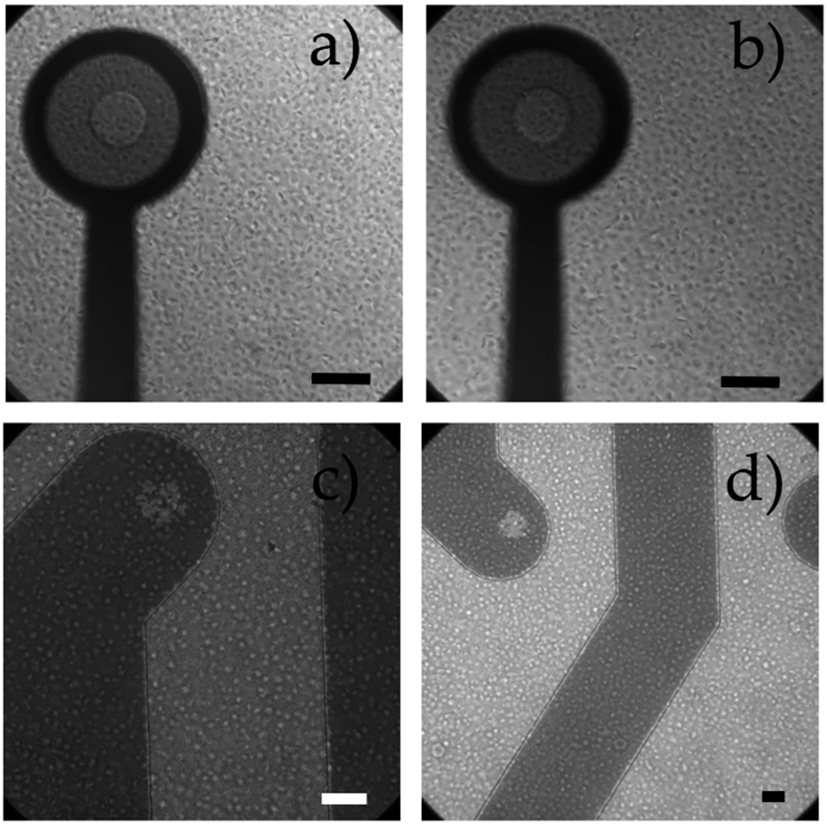

Figure S4. SM—DIC microscopy images of platelets distributed homogeneously in the SSP+ medium over microelectrodes and nearby on the BDD-on-quartz and BDD-on-glass devices. Upper, the BDD-on-quartz MEA. **Lower,** the BDD-on-glass MEA. All images of platelets were taken at a concentration of $4{\cdot}10^5/\mu L$. Calibration bars = 20 μm.

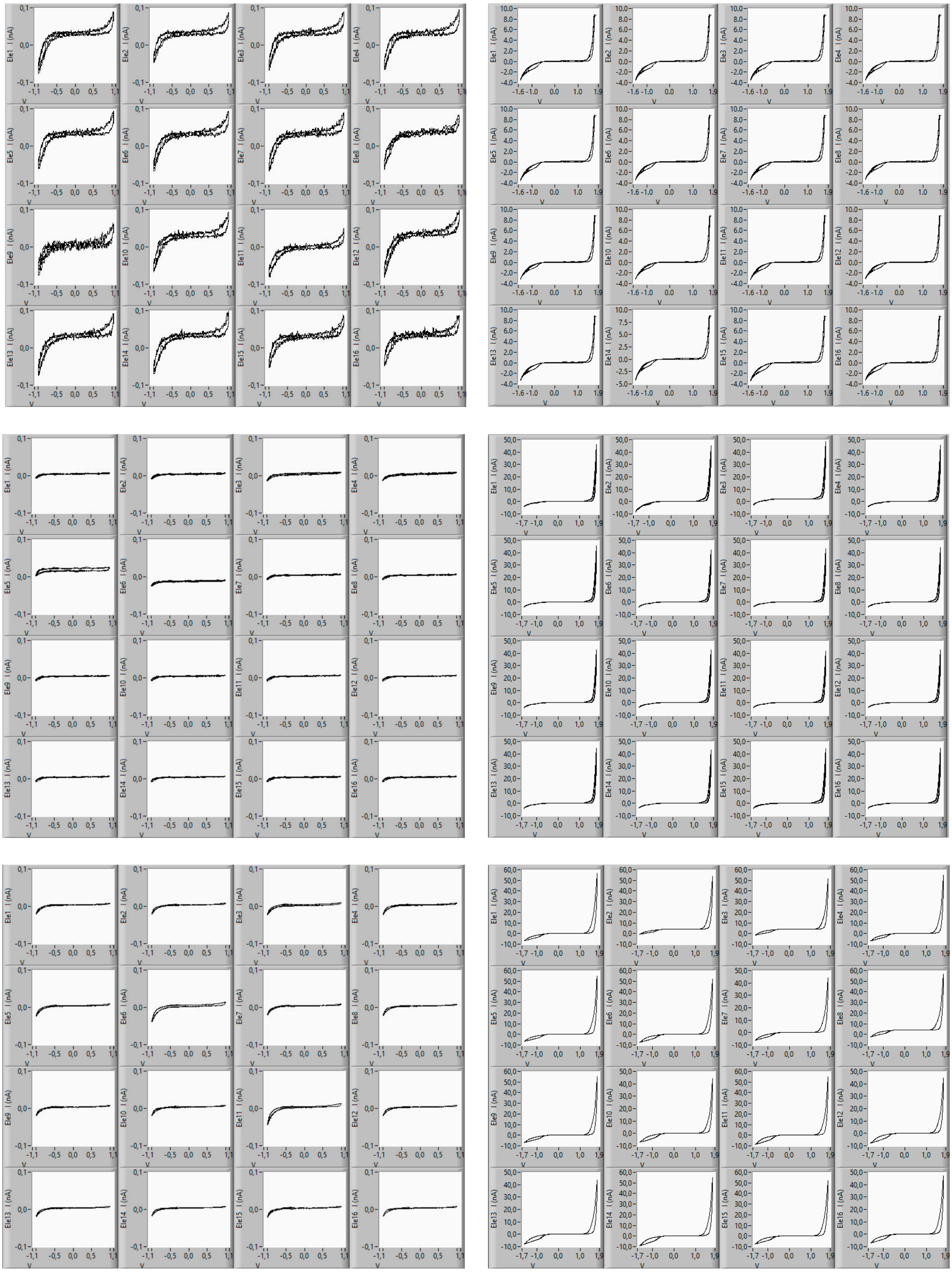

Figure S5. SM—Cyclic voltammograms from the three MEA devices. All CV plots from the 16-channel MEAs are recorded over a bandwidth of 10 Hz at a scan rate of 200 mV/s in 100 mM KCl. **Upper panels,** the BDD-on-glass MEA. **Medium panels,** the BDD-on-silicon MEA. **Lower panels,** the BDD-on-quartz MEA. The background current in the absence of redox activity

corresponding to the flat portion of the CV plots, approximately between -0.8 V and +1 V, can be appreciated in the left panels. The electrode activity can be observed by scanning over a larger potential range, thus reaching the reduction and oxidation currents corresponding to hydrogen evolution and oxygen evolution, respectively, due to water splitting. BDD-on-glass shows higher noise and background currents and lower activity than both the BDD-on-silicon MEA and the BDD-on-quartz MEA, which are very similar.

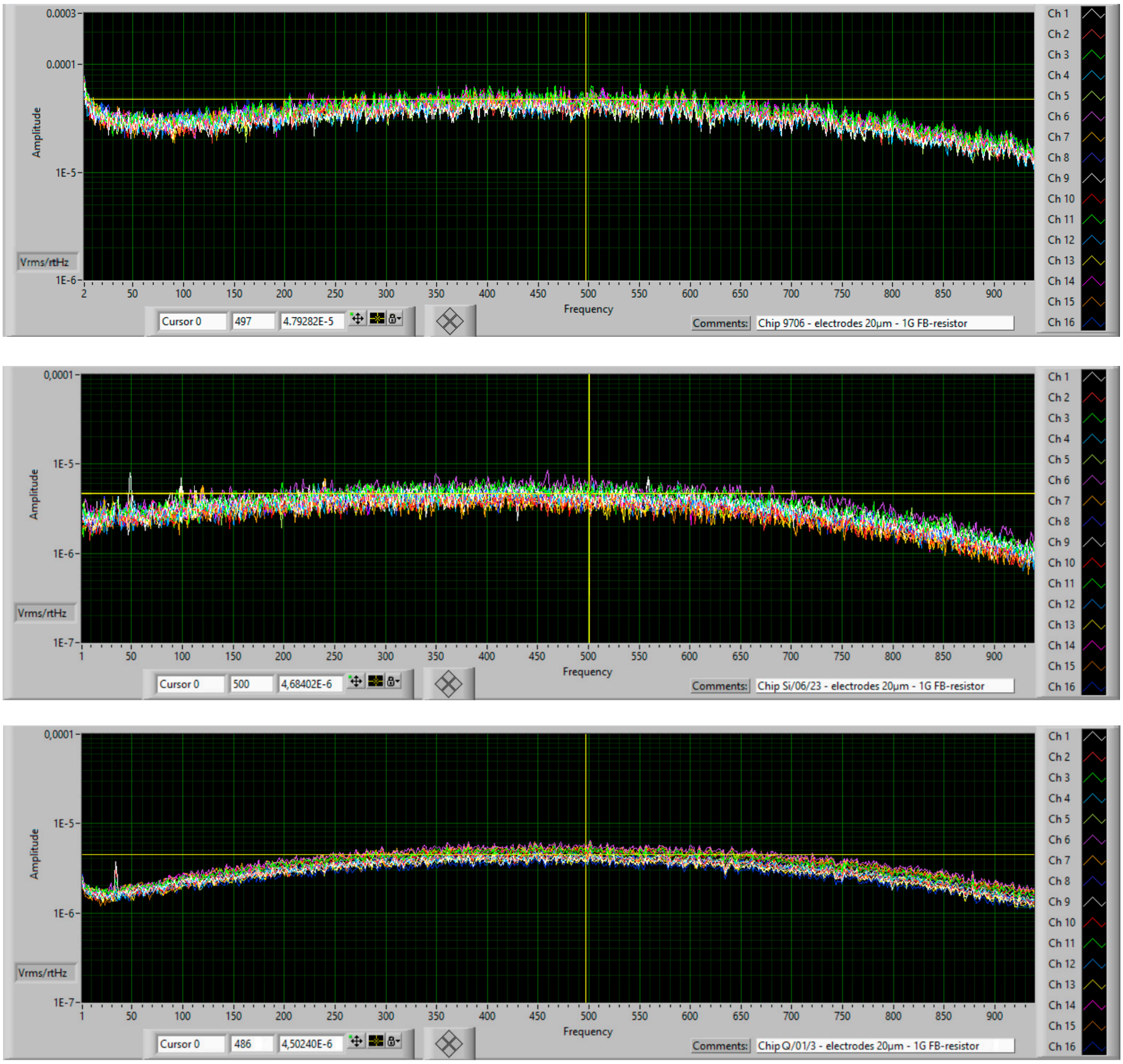

Figure S6. SM—Noise spectra. Upper, the BDD-on-glass MEA. **Medium,** the BDD-on-silicon MEA. **Lower,** the BDD-on-quartz MEA

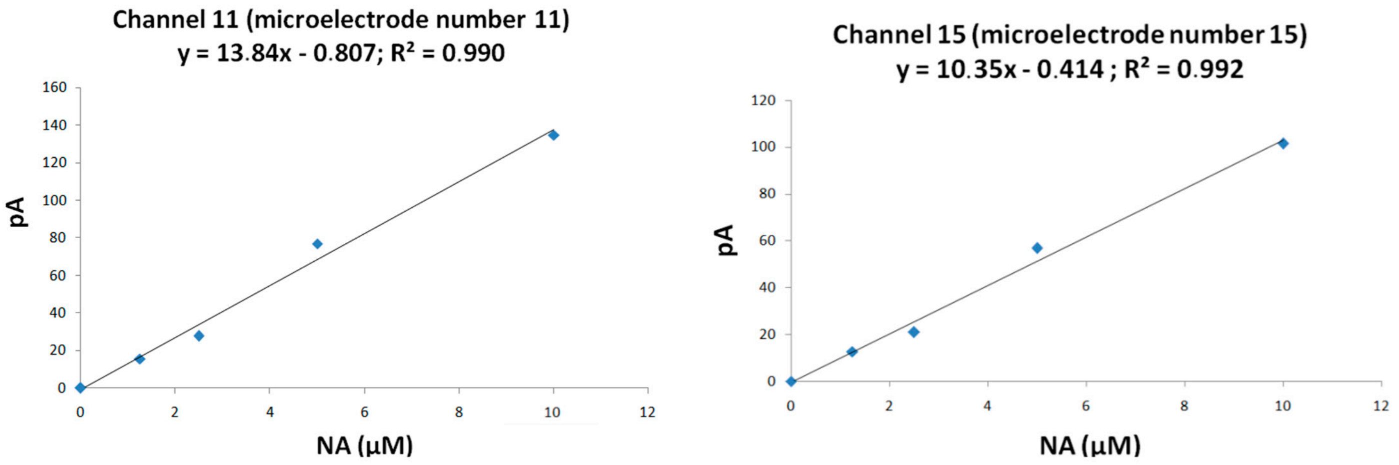

Figure S7. SM—Calibration of two channels of the same BDD-on-quartz MEA device obtained from the graphical representation of the current intensities recorded at different final norepinephrine (NA) concentrations in SSP+ (0; 1.25; 2.5; 5.0; and 10.0 NA µM). Recordings were made at a potential of +800 mV. The LabVIEW program was used for data acquisition, and IGOR-Pro software was used for the quantitative analysis of the recorded currents. **Right,** the calibration line of measurements recorded on microelectrode number 11. **Left,** the calibration line of the results determined on microelectrode 15.

Disclaimer/Publisher's Note: The statements, opinions and data contained in all publications are solely those of the individual author(s) and contributor(s) and not of MDPI and/or the editor(s). MDPI and/or the editor(s) disclaim responsibility for any injury to people or property resulting from any ideas, methods, instructions or products referred to in the content.
